# High Genetic Diversity in a Potentially Vulnerable Tropical Tree Species Despite Extreme Habitat Loss

**DOI:** 10.1371/journal.pone.0082632

**Published:** 2013-12-18

**Authors:** Annika M. E. Noreen, Edward L. Webb

**Affiliations:** Department of Biological Sciences, National University of Singapore, Singapore; Manchester Institute of Biotechnology, United States of America

## Abstract

Over the last 150 years, Singapore’s primary forest has been reduced to less than 0.2% of its previous area, resulting in extinctions of native flora and fauna. Remaining species may be threatened by genetic erosion and inbreeding. We surveyed >95% of the remaining primary forest in Singapore and used eight highly polymorphic microsatellite loci to assess genetic diversity indices of 179 adults (>30 cm stem diameter), 193 saplings (>1 yr), and 1,822 seedlings (<1 yr) of the canopy tree *Koompassia malaccensis* (Fabaceae). We tested hypotheses relevant to the genetic consequences of habitat loss: (1) that the *K. malaccensis* population in Singapore experienced a genetic bottleneck and a reduction in effective population size, and (2) *K. malaccensis* recruits would exhibit genetic erosion and inbreeding compared to adults. Contrary to expectations, we detected neither a population bottleneck nor a reduction in effective population size, and high genetic diversity in all age classes. Genetic diversity indices among age classes were not significantly different: we detected overall high expected heterozygosity (H_e_ = 0.843–0.854), high allelic richness (R = 16.7–19.5), low inbreeding co-efficients (F_IS_ = 0.013–0.076), and a large proportion (30.1%) of rare alleles (i.e. frequency <1%). However, spatial genetic structure (SGS) analyses showed significant differences between the adults and the recruits. We detected significantly greater SGS intensity, as well as higher relatedness in the 0–10 m distance class, for seedlings and saplings compared to the adults. Demographic factors for this population (i.e. <200 adult trees) are a cause for concern, as rare alleles could be lost due to stochastic factors. The high outcrossing rate (t_m_ = 0.961), calculated from seedlings, may be instrumental in maintaining genetic diversity and suggests that pollination by highly mobile bee species in the genus *Apis* may provide resilience to acute habitat loss.

## Introduction

Deforestation and degradation of primary forests are of critical concern for global biodiversity conservation [1]. The island city-state of Singapore (704 km^2^, population 5.18 million) is one of the most extreme examples of urbanization in the world [2], [3], [4]. Until the mid-1800’s the island was predominantly primary lowland rainforest (∼80%) with additional substantial areas of mangrove and freshwater swamp forest [5]. Rapid deforestation beginning ∼150 years ago has reduced overall forest cover to less than 2% of its original extent: 118 ha (0.16%) of primary and 995 ha (1.37%) of old secondary forest [2], [3].

In addition to outright extirpation following habitat reduction, extinctions of local flora and fauna may continue after initial deforestation, as a subset of species within small and isolated habitat remnants may continue to decline in response to stochastic, demographic and genetic factors, i.e. an extinction debt [6], [7]. In line with these predictions, Singapore’s native flora and fauna has been affected by “catastrophic” extinction rates [8], and the continuing loss of native, forest-dependent species remains a concern [3], [9]. Detrimental genetic effects from a reduction in population size can include genetic erosion (i.e. loss of rare alleles and lower genetic diversity), which reduces the genetic variation for selection to act upon [10], [11], and higher inbreeding. These changes may be particularly important for outcrossing species [12], [13], as they can significantly affect fitness, fertility, and offspring viability, as shown by studies of laboratory [14], captive [15], and wild [16] populations.

However, interpreting genetic diversity indices in relation to population viability can depend on phylogenetic history, life history and biogeographic variables. For example, some plant species that are naturally rare and/or endemic have strategies that allow for survival under conditions of self-fertilization or clonality, low genetic diversity, and high inbreeding [17], [18]. However, widespread, abundant, and outcrossing species may lack mechanisms allowing persistence if their populations undergo precipitous declines. Meta-analyses have concluded that previously common tree species that undergo fragmentation and isolation are more susceptible to the negative genetic consequences of deforestation than naturally rare species [19], with “strong, negative and significant effects” on genetic diversity [20]. These consequences may be apparent only after several generations (i.e. >100 years) for some long-lived species [19], [20], [21], and a recent meta-analysis has concluded that woody species are equally prone to loss of genetic diversity compared to other plant species [22]. Indeed, several recent studies have documented a significant decline in genetic diversity in tropical tree species immediately following habitat loss and fragmentation [23], [24], [25]. Additionally, increasing spatial genetic structure (SGS) intensity in younger cohorts has recently been used to document a negative genetic effect of habitat loss and fragmentation on a tropical forest tree despite a lack of genetic diversity reduction [26], although demographic factors relating to age cohorts may also play a significant role in interpreting spatial genetic structure [27].

Immigration and gene flow into small, isolated populations is generally considered to have positive genetic and demographic effects, as gene flow can increase effective population size and genetic diversity, and reduce deleterious inbreeding, e.g. [16], [28]. For Singapore, the next-nearest primary forests are more than 70 km distant (on peninsular Malaysia) and separated by the Singapore Strait as well as urban and agricultural landscapes. Hence, geographic isolation likely corresponds to genetic isolation, as naturally occurring dispersal (of either pollen or seed) from outside populations is improbable. Thus, in the absence of human-mediated migration (e.g. transplants) Singapore’s primary forest species may be fully dependent on recruitment from local individuals for persistence.

### Target species

*Koompassia malaccensis* Maingay *ex* Benth. (Fabaceae) is a long-lived (up to several hundred years), large (up to 55 m tall), invertebrate-pollinated rainforest tree with wind-dispersed seeds. It is a canopy emergent tree of primary rainforest in Southeast Asia, ranging from Thailand to Borneo. Despite being classified as a species of low extinction risk over its entire range [29], *K. malaccensis* is locally endangered within Singapore owing to severe habitat loss [9]. As a commercially valuable timber species, *K. malaccensis* has been widely harvested and therefore tends to be relegated to primary forest patches or occasionally found as isolated remnant adults in secondary forests. In some areas individual trees are left intact as *K. malaccensis* is frequently utilized by wild honey bees (*Apis dorsata* in particular) for nesting [30]. Pollination of *Koompassia* is accomplished by *A. dorsata*, which is a large, migratory bee species, along with other *Apis* species (R.T. Corlett, pers. comm.). As a member of the Fabaceae, reproduction is hypothesized to occur yearly [31], although observations suggest that seed set in Singapore might be more sporadic (E.L. Webb and S.K.Y. Lum, pers. obs.). *K. malaccensis* is fast growing, and reaches reproductive maturity at around 30 cm stem diameter (perhaps 30–40 years old assuming a 1 cm annual diameter increment [32]).

Previous research on *K. malaccenis* has been conducted on peninsular Malaysia, Singapore’s next-nearest primary forest populations [33], [34], [35]. Across 19 populations throughout peninsular Malaysia, *K. malaccensis* was found to have high genetic diversity and low differentiation [34]. A Malaysian population also showed low biparental inbreeding and a predominately outcrossing mating system [35], which is in accordance with other tropical tree species. Being the closest populations to Singapore, these Malaysian *Koompassia* populations can be used as an approximate baseline population, although direct comparisons should be undertaken with caution.

Although no pre-deforestation abundance and distribution data for *Koompassia* or any other species exist for Singapore, it is known that Singapore was predominately lowland tropical rainforest (∼80%) [5]. As the current distribution of *K. malaccensis* is almost exclusively in primary forest patches, it is fair to assume a high correlation between primary forest habitat loss and population loss. Thus we estimated that owing to habitat loss, *K. malaccensis* has sustained a population reduction of at least 90% over the last >150 years. It is important to note that whether actual population loss is exactly equivalent to the reduction of primary forest area is somewhat inconsequential given the scale of deforestation [2], [3]. A previous study on the effect of population reduction of *K. malaccenis* through logging in southern peninsular Malaysia —a disturbance that typically removes a lower proportion of a tree population than deforestation—found fewer alleles per locus for seedling, sapling, and adult *K. malaccensis* individuals in logged versus unlogged plots, as well as a reduction in the number of polymorphic loci [33]; however, low sample sizes in that study prevented definitive conclusions. Given that previous research over smaller spatial and shorter temporal scales showed genetic consequences of logging on *K. malaccensis*, a study of the effects of extreme habitat loss in Singapore was well justified.

### Objectives

*Koompassia malaccensis* in Singapore is a putatively previously abundant, widespread and outcrossing species that underwent a severe decline in habitat starting ∼150 years ago and has a low likelihood of immigration and gene flow from outside populations. Hence, we tested two hypotheses relevant to the genetic consequences of habitat loss. First was that the *K. malaccensis* population in Singapore experienced a population bottleneck and a reduction in effective population size. Second was that recruit cohorts would show genetic erosion (i.e. lower allelic richness and/or heterozygosity) and inbreeding compared to the adult cohort. To test these hypotheses, we assessed the genetic structure of comprehensively sampled adults, and randomly selected samples of saplings and seedlings, using eight highly polymorphic microsatellite loci.

## Materials and Methods

**Figure 1:**
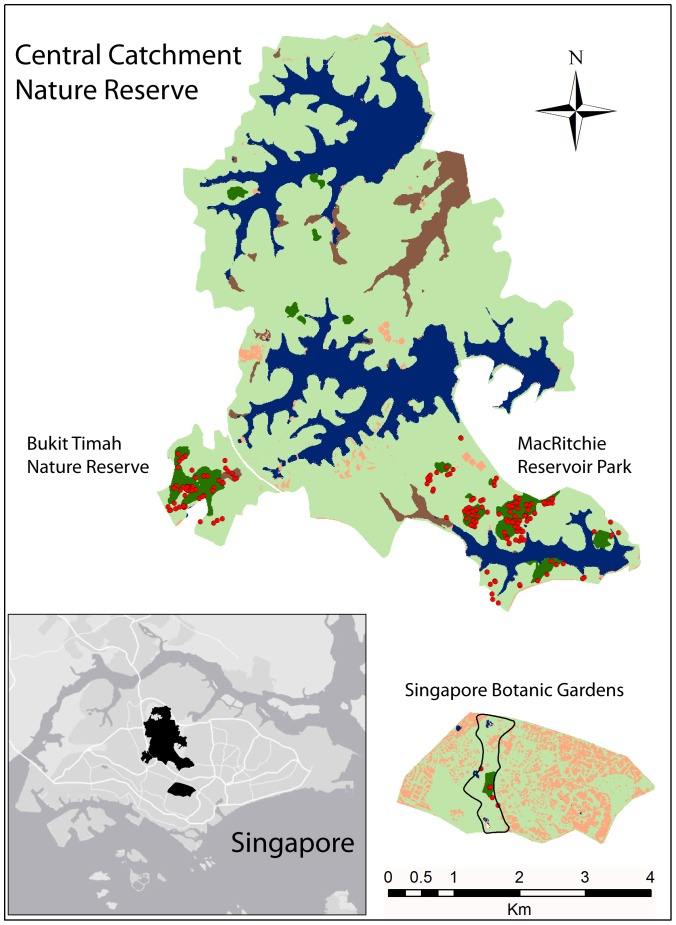
Map of Singapore’s remaining primary forest fragments within a secondary forest and urban matrix. Dark green – primary forest; Light green – secondary forest; red dots – adult *K. malaccensis* individuals.

Our study area encompassed the central catchment and the Singapore Botanic Gardens (Figure 1). The central catchment, a mosaic of primary and secondary forest patches set within an urban matrix, consists of the Central Catchment Nature Reserve (CCNR) complex (MacRitchie, Lower Pierce, Upper Pierce and Upper Seletar Reservoir forests) and Bukit Timah Nature Reserve (BTNR), and accounts for the largest area of contiguous forest remaining in Singapore: ca. 3,043 ha (primary, old secondary and young secondary forest), of which 2880 ha is in CCNR and 163 ha in BTNR [36] (Figure 1). From November 2010 through November 2011, we conducted fieldwork to locate and survey all *K. malaccensis* adults with a dbh (stem diameter at 1.4 m above the base) of at least 30 cm in BTNR and MacRitchie Reservoir Park. For BTNR, we were provided access to an existing spatial database of all trees >30 cm dbh, from which we extracted the locations of all adult *K. malaccensis*. MacRitchie surveys were conducted by referring to existing vegetation maps of the central catchment [2], [5] and taking extensive reconnaissance hikes through primary as well as secondary forest to locate adults. The Botanic Gardens retains five *K. malaccensis* adults, all of which we sampled. Our data set was collected in >95% of the remaining primary forest area in Singapore, and was therefore expected to account for a roughly equivalent percentage of the remaining *K. malaccensis* population. However, given that we found a few isolated adults located in secondary forest, it is possible that we captured a somewhat smaller proportion of the remaining population than 95%.

### Sample collection

We collected bark and/or leaves of 179 adult *K. malaccensis* (dbh >30 cm) and leaves from 193 saplings (>1 year old, 55 cm to 2+ m height) and 1,822 seedlings (<1 year old, ≤50 cm, often with cotyledons still attached) from beneath 122 adults. Sampling was conducted under Singapore National Parks research permit NP/RP10-078. All necessary permits were obtained for the described study, which complied with all relevant regulations.

Our field observations of isolated adults (i.e. >50 m to the next nearest *K. malaccensis*) indicated that most recruits occur within 10 m of the parental tree. All recruits sampled had their compass bearing and distance measured to the nearest adult *K. malaccensis*, which was designated the putative mother. For each recruit, 3 to 5 leaflets were collected, placed in labelled bags and dehydrated with silica gel. We used one or both of the following sampling methods for adults: if no other adult *K. malaccensis* tree was within 50 m, a freshly fallen leaf was collected directly under the crown. If other adults were within 50 m, a 1 cm×1 cm bark scraping (<4 mm deep) was taken using sterilized equipment, placed in a 1.5 mL Eppendorf tube, and stored at −20°C the same day.

Sample DNA was extracted using a modified CTAB-chloroform extraction [37] using an OMNI bead disruptor for initial tissue lysis. DNA samples are stored in TE at −20°C.

### Microsatellite amplification and genotyping

Eight highly robust microsatellite markers [38] (Table 1) were fluorescently labelled (VIC, FAM, NED, or PET: Applied Biosystems, Carlsbad, CA, USA) and grouped into two multiplex reactions of four markers each. PCR conditions for both multiplexes were as follows: 0.25 unit *Taq* polymerase and 0.5 unit buffer (New England BioSciences, Ipswich, MA, USA), 20 to 80 picomol of each F- and R- primer; 5 mM dNTPs, 2.5 mM MgCl_2_ and 1% DMSO final concentration in 5 µl total volume reactions. Cycling conditions: 95°C for 1.5 minutes; 34× 95°C for 30 seconds, 56°C for 30 seconds, 72°C for 30 seconds, and a final 5 minute 72°C extension. For each sample, the two multiplex PCR reactions were pooled for a single fragment analysis (ABI 3100 or 3730x) and run against a 600-LIZ internal size standard (ABI). Samples were scored using GeneMapper v. 4.1. One-third of adults and 0.7% of the recruit cohorts were re-extracted, PCRed and genotyped in their entirety, and 100% of adults and 24.9% of the recruit cohorts were re-PCRed and genotyped at 1 to 8 loci to confirm rare alleles as well as to estimate genotyping errors. Genotyping errors were calculated per locus as the percent of non-matching alleles in a second PCR (Table 1).

**Table 1:** Sample size (N) per locus, size range of alleles, total number of alleles detected, observed (H_o_) and expected (H_e_) heterozygosity, genotyping error rate, percent missing data, percent null alleles, and inbreeding co-efficient values (F_IS_).

Locus	N	Size range	# alleles	H_o_	H_e_	Typing error	Missing data	Null alleles	F_IS_
Km011	2190	162–188	13	0.601	0.635	0.1%	0.1%	-	0.054
Km082	2185	231–275	22	0.863	0.897	0.2%	0.3%	-	0.038
Km158	2187	226–268	20	0.774	0.851	0.2%	0.2%	-	0.091
Km180	2188	236–269	20	0.846	0.873	0.1%	0.2%	-	0.031
Km071	2098	160–206	23	0.447	0.909	0.3%	**4.3%**	∼**30%**	**0.508**
Km127	2187	142–184	18	0.881	0.894	0.1%	0.2%	-	0.014
Km141	2163	293–331	21	0.785	0.903	0.3%	1.3%	∼5%	0.131
Km143	2184	315–351	19	0.761	0.861	0.4%	0.8%	∼5%	0.117

Bold indicates significant null alleles.

### Data analysis

Microchecker v. 2.2.3 [39] was used to detect the presence of null alleles as well as possible technical scoring errors. Given the high levels of null alleles detected in locus Km071, this locus was excluded from the calculations of F_IS_ values (the inbreeding co-efficient, used to detect inbreeding), as the conclusions drawn would not be biologically accurate—i.e. a high F_IS_ value at this locus would not reflect inbreeding. To evaluate the adequacy of our field sampling in capturing the full range of allelic diversity in Singapore, we produced curves for each locus depicting the accumulation of alleles as a function of the number of samples analyzed.

Allelic richness (R), expected and observed heterozygosity (H_e_, H_o_) and inbreeding co-efficients (F_IS_) were calculated with Fstat v. 2.9.3 [40]. H_e_ and R among age classes were compared with an Analysis of Variance at the locus level. For mating system calculations, seedlings or saplings were classified as the progeny of the nearest adult *K. malaccensis*. Mltr v. 3.4 [41] was used to calculate outcrossing rates (t_m_) and biparental inbreeding rates (t_m_-t_s_) under default parameters and with the possibility of null alleles; the putative maternal genotype for each set of putative offspring was included. Four chosen loci (Km011, Km127, Km158a, Km180) were analysed for a random subsample of 9 adults in order to compare the mltr results from the Singapore population to the Malaysia population under more analogous parameters.

M_P_val v. 2 [42] was used to detect a potential reduction in effective population size in the adult as well as recruit cohorts, as theory predicts that a population reduction of 50% is expected to lead to a 20% loss of discrete allele sizes and would correspond to gaps in the allele size distribution [42]. An M-ratio value <0.70 generally indicates that a population has undergone a reduction in effective population size, whereas a population with an M-ratio value >0.80 indicates population stability. The program Bottleneck [43] utilizes the assumption that rare alleles will be lost more quickly than heterozygotes during a bottleneck, and detects a mode-shift under conditions consistent with a recent bottleneck. Depending on the initial population size, the decay in the genetic signature of bottleneck detection under random mating means that bottlenecks can usually be detected no more than 2 to 3 generations prior to the current generation, whereas M_P_val can be used to detect more historical population size reductions. Bottleneck v. 1.2.02 was run for adult, juvenile and seedling populations respectively, using the infinite allele and stepwise mutation models with 10,000 iterations per run.

Spatial genetic structure (SGS) for the adults, saplings, and seedlings was analyzed with GenAlex v. 6.5.3 [44] under the following parameters: 999 permutations and 999 bootstraps, user-defined distance classes (5, 10, 20, 40, 80, 160, 320, 640, 1280, 2560, and 5120 m)—except no 5 m distance class was possible for the adult cohort due to too few individuals within this distance class. Significance of autocorrelogram results (testing both the null hypothesis of no structure for each cohort as well as significant differences between cohorts) was determined using the “Multiple pops” option using the above parameters and the adult cohort distance classes (i.e. 0–10 m for the first distance class). The relatedness metric “r” (Queller & Goodnight) [45] was also obtained from GenAlex; full siblings have r-values of 0.50 and half siblings, 0.25. SpaGeDi v. 1.4 [46] was used to determine spatial genetic structure intensity (S_p_) using the formula -b/(1-F_1_), where b is the slope of the regression between pairwise kinship coefficients [47] and the logarithm of spatial distance, and F_1_ is the mean pairwise kinship coefficient within the first distance class (see [48]). Identical (adult) distances classes were entered for all three cohorts. Approximate 95% confidence intervals for S_p_ were calculated as±2× the standard error of b [49]. Calculations were performed with 9999 permutations and jackknifing over loci.

## Results

**Figure 2:**
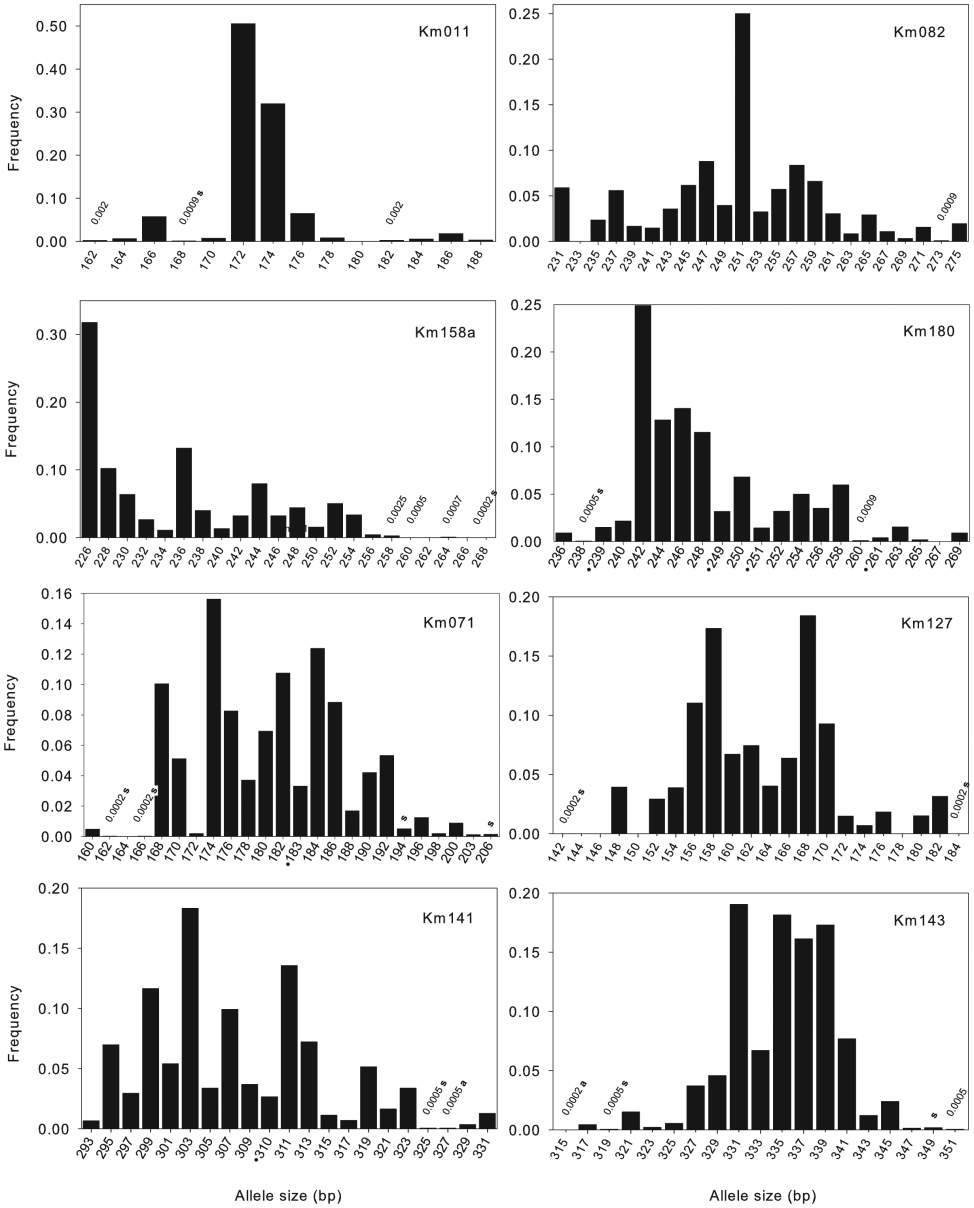
Allele frequencies for each locus. Alleles present in very low numbers have the frequency presented above the allele if the bar too small to be easily visualized. All potential allele sizes within the captured range (assuming normal dinucleotide repeat units) are included in the x-axis; blanks indicate that this allele size was not captured. A dot below the allele indicates an unusual size (1 bp difference from nearest allele). ‘a’ indicates a private allele in the adult age class, ‘s’ indicates a private allele in the seedling cohort.

Genetic diversity was high and not significantly different between age classes (H_e_ = 0.843–0.854, p = 0.98; R = 16.72–19.45, p = 0.20) (Table 2). The inbreeding co-efficient values for all three age classes were low (F_IS_ = 0.013–0.076) and not significant (p = 0.18), although there was an upward trend in younger age classes (Table 2). There were twelve private alleles in the seedling cohort and two private alleles in the adult cohort (Figure 2). Over the whole dataset, the multilocus outcrossing rate was high (t_m_ = 0.961, SD 0.015) and the biparental inbreeding rate (t_m_-t_s_) was 0.111 (SD 0.011) (Table 2).

**Table 2:** Comparisons of genetic diversity, reproductive, and spatial genetic structure indices among Singapore *K. malaccensis* cohorts with reference to Malaysian populations [34], [35].

	Singapore^1^			Malaysia
	Seedling	Sapling	Adult	Adult
	n = 1,822	n = 193	n = 179	n = 456
H_e_	0.853	0.843	0.854	0.798^3^
R	19.45	16.72	17.88	9.70^3^
^2^F_IS_	0.076	0.037	0.013	
r (0–10 m)	0.285^a^	0.305^a^	0.132^b^	
S_p_	0.0287 (0.0251–0.0323)	0.0131 (0.0075–0.0187)	0.0044 (0.0030–0.0058)	
	M_s_ 0.957		M_a_ 0.857	
1t_m_: 0.961 (0.01)				
1t_m_-t_s_: 0.111 (0.011)				
^4^t_m_: 0.923 (0.074)				^4^t_m_: 0.890 (0.041)
^4^t_m_-t_s_: 0.018 (0.053)				^4^t_m_-t_s_: 0.026 (0.013)

_e_, mean expected heterozygosity; R, mean allelic richness; F_IS_, mean inbreeding co-efficient excluding locus Km071 (frequency null alleles ∼0.30); r (0–10 m), average relatedness (r, Queller & Goodnight 1989) for the 0–10 m distance class; S_p_, spatial genetic structure intensity (approximate 95% confidence intervals); M, M-values for adult cohort (M_a_), and for the seedling plus sapling cohorts combined (M_s_); t_m_, outcrossing rate and t_m_-t_s_, biparental inbreeding rates (standard deviations in parentheses). ^a^ and ^b^ indicate significant differences between age classes (p<0.05). H

^2^seven loci; ^3^six loci, ^4^4 loci and 9 adults.^1^ eight loci;

**Figure 3:**
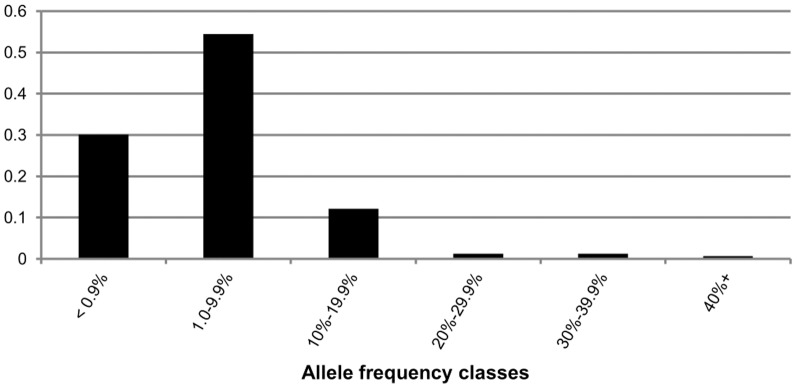
Frequency of different allele frequency classes.

**Figure 4:**
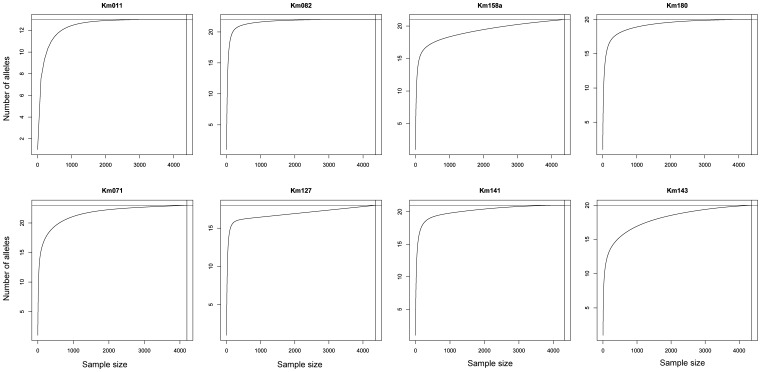
Allelic richness accumulation curves for each locus. Rarefaction curves were produced in the R statistical environment [69].

We detected neither a genetic bottleneck in any age class (no mode shift) nor a reduction in effective population size (M-ratio = 0.857 and 0.957 for the adult and recruit cohorts, respectively) (Table 2). The large number of rare alleles and the few allele size gaps in most loci support this conclusion (Figure 2). Rare alleles (<1%) were most common in tails of the size distribution for most loci (Figure 2) and comprised 30.1% of the total data set (Figure 3). Allele accumulation curves (Figure 4) indicated that we likely captured the majority of the alleles present at these loci in *K. malaccensis* within Singapore.

**Figure 5:**
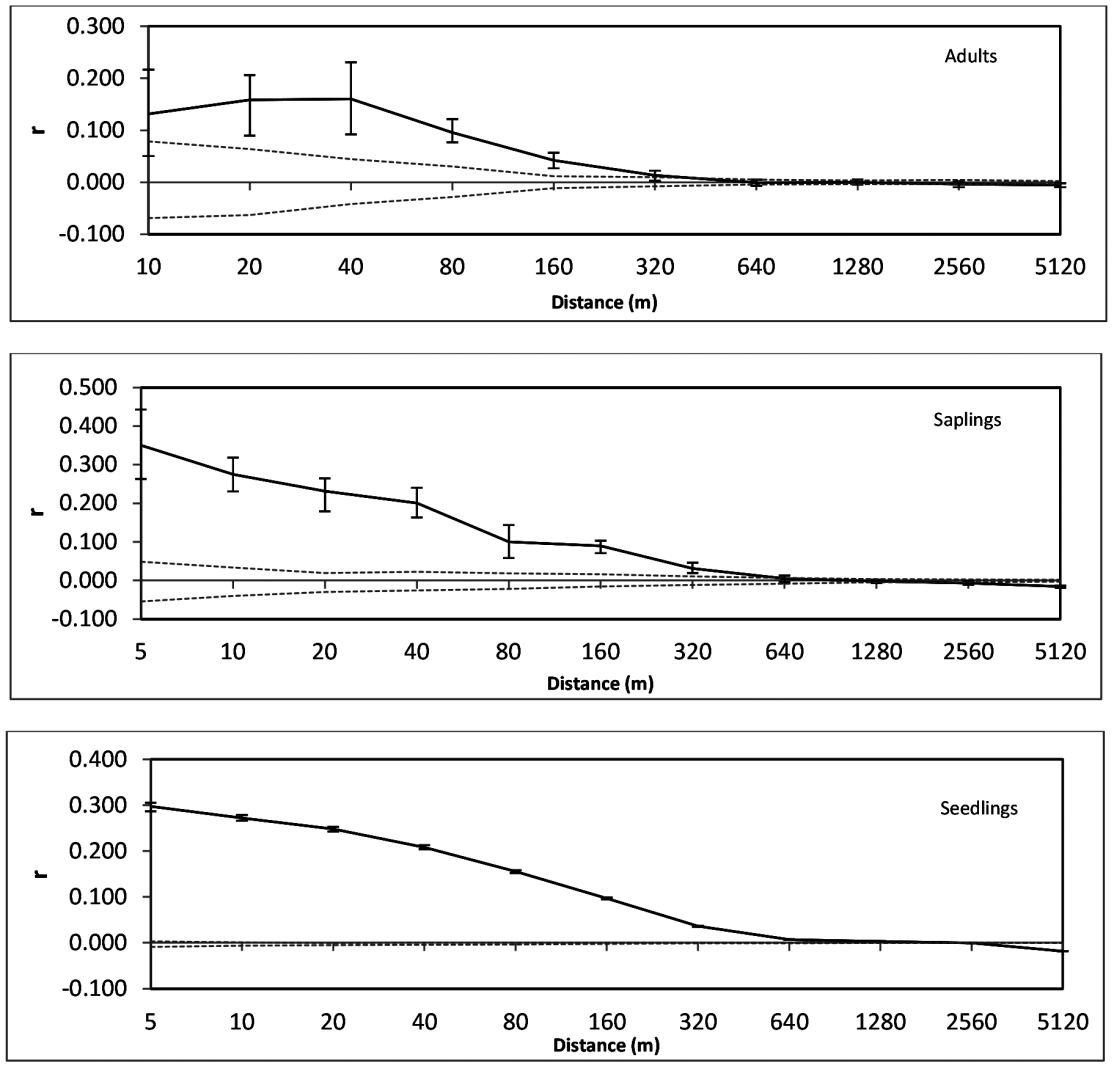
Spatial genetic structure autocorrelograms for adults, saplings, and seedlings, respectively. ‘r’ is the relatedness coefficient of Queller & Goodnight [45] as implemented in GenAlex. Error bars are 95% confidence intervals for the value of r. Upper and lower bounds (dashed lines) represent the 95% confidence intervals for the null hypothesis (no spatial structure).

All three cohorts had spatial genetic autocorrelograms that deviated significantly from the null hypothesis, i.e. all had significant genetic structure (p<0.001 for all) (Figure 5). The SGS of the adult cohort was significantly different from the seedling and sapling cohorts (p<0.001), although the seedling and sapling cohorts were not significantly different (p = 0.30). In addition, the overall mean relatedness (r) at the 0–10 m distance class for the adult cohort was approximately half that of the recruit cohorts; this difference was significant between adults and the seedling and sapling cohorts (p = 0.021 and 0.029, respectively) although not between the seedlings and saplings (p = 0.36) (Table 2). S_p_ was lowest in the adult cohort (0.0044), and increased with younger cohorts (saplings = 0.0131, seedlings = 0.0287) (Table 2). The non-overlapping confidence intervals indicate significant differences in S_p_ between each of the three age classes.

## Discussion

Gene flow and intergenerational studies using exhaustively surveyed landscapes are crucially lacking in the tropical tree literature [50]. In the few recent studies using comprehensive surveys (i.e. >95% of the population likely captured) for formerly widespread tree species, significant reductions in genetic diversity were most often detected in the recruit cohort [22], [23], [24]. Thus, our results stand in contrast to theoretical predictions as well as many experimental studies of the genetic consequences of past deforestation (reviewed in [22]). This study detected neither a genetic bottleneck nor a reduction in effective population size, as well as high genetic diversity and a large proportion of rare alleles in all age classes. Genetic studies of *K. malaccensis* in Malaysia [33], [34], [35] provide some reference points (Table 2) and indicate that genetic diversity of *K. malaccensis* in Singapore is generally on par with populations studied in more intact Malaysian forests. While these unexpected results may not be representative of the majority of primary forest species (and demographic factors are still a cause for concern) they demonstrate lower than expected genetic vulnerability for a locally threatened, primary forest tree species in a highly urbanized landscape.

### Genetic diversity and population viability

The initial decline in genetic diversity during habitat loss is due to demographic factors, i.e. the removal of individuals. Subsequently, genetic drift and reduced genetic diversity generally results from only a subset of individuals contributing gametes to the next generation, e.g. [25], [51]. However the genetic diversity detected for *K. malaccensis* in Singapore would be considered high even for common, widespread species in pristine habitats [21] and there were no significant differences in genetic indices between age classes. We captured a large fraction of all alleles based on the accumulation curves (Figure 4): alleles with a frequency of less than 1% constituted 30.1% of the data set, and 84.6% of the total number of alleles had a frequency of less than 10% (Figure 3). In addition, there were few gaps in the size distribution (Figure 2), which is contrary to theoretical expectations as well as experimental studies that have documented a loss of alleles during severe population decline [52], [53].

The majority (∼99%) of alleles present in the adult population were also present in the recruit cohort, in addition to twelve private alleles in the seedling cohort (Figure 2). Given that all private alleles were PCRed at least twice, genotyping errors were unlikely to have contributed substantially to these results. Explanations for these rare, putatively private alleles in the seedlings could include the following: 1. Unsampled adult trees containing these rare alleles within the existing primary forest fragments; 2. Gene flow from isolated trees containing these rare alleles located within smaller forest fragments in the intervening landscape or remnant trees in Singapore’s urbanized matrix; 3. Spontaneous mutations, which could account for some of the private alleles given that ∼2,000 seedlings were genotyped, and experimental mutation rates for dinucleotide repeats in plant species is estimated to be ∼2.4×10^−4^ mutations/generation/locus [54].

Low inbreeding and high heterozygosity are positive indicators for short-term population viability; high allelic richness and a large proportion of rare alleles indicate an adequately large effective population size for long-term viability [55]. While only a relatively small number of individuals may be needed in the short term to prevent inbreeding, greater genetic variation is thought to increase the likelihood of survival over much longer time scales [11]. Genetic diversity measured via neutral microsatellite markers is likely correlated to genetic variation at functional genes [56], and adaptation or resilience to novel or changing habitats is most often positively correlated to genetic variation [57], [58]. Thus, management strategies should be implemented that maintain as far as possible the full complement of genetic diversity within Singapore.

In the present study, the adult cohort had low SGS intensity (S_p_ = 0.0044) similar to other tropical tree species (see review of S_p_ values for trees in [59]). This corresponds to recent research showing that long-lived tropical rainforest canopy tree species pollinated by the highly mobile, migratory bee species *Apis dorsata* exhibited less SGS compared to species pollinated by smaller, less mobile pollinators [60]. Thus, a signature of frequent long-distance pollen dispersal may remain in the adult cohort. In contrast, the recruit cohorts had significantly different SGS autocorrelogram structure compared to the adult cohort and significantly higher mean relatedness in the 0–10 m distance class. Moreover, SGS intensity significantly increased as cohorts got younger (Table 2). While the results provide only a one-time snapshot of the spatial genetic structure, they could point to one of two trends. First, the results could indicate that recruit cohorts of *K. malaccensis* are showing sensitivity to habitat loss (e.g. [26]). If this was the case, S_p_ and mean relatedness in the recruit cohorts would remain comparatively higher than the present adult cohort as they mature. Alternatively, given that the majority of seedlings and saplings die before reaching maturity, the present-day recruit cohorts may converge towards adult SGS intensities as their numbers undergo thinning (e.g. [27]). Long-term population monitoring would be necessary to determine which explanation is most fitting.

The overall outcrossing rate (t_m_) for the whole dataset as well as the subset was as high as in the Malaysian populations [34]. However, it is possible that our sampling strategy (see Methods) could have led to a slight inflation of outcrossing rate for the Singapore population. In contrast, the biparental inbreeding rate (t_m_-t_s_) was much higher overall in Singapore than in Malaysia (Table 2), indicating that trees with higher relatedness are mating at a greater frequency compared to the Malaysian population. While this interpretation would not be unexpected given our other results, there could be confounding variables that reduce the validity of this comparison. In particular, the spatial distance between the trees in the Malaysia study was not reported (they were “distant” from each other [34]), and hence the difference in values could simply reflect that a larger number of adjacent adult trees were sampled in Singapore, with the likely consequence of detecting matings between more closely related neighbours. This speculation is supported by the results of a random subsample of 9 Singapore adults (analyzed using only 4 loci, one of which was the same between studies), in which the biparental inbreeding estimates were on par with the Malaysian population (Table 2). Regardless of comparisons to Malaysia, the overall data from Singapore showed a high level of outcrossing, but the moderate level of outcrossing occurring between related adults could ultimately increase the level of inbreeding.

### Ecology, demographics, and population viability

Once a population reaches a critically low threshold of individuals, demographics can play an equal—if not more important—role in long-term population persistence compared to genetic factors, although the two are often inexorably linked [57], [61]. A previous study estimating a universal minimum viable population size of ≈5,000 [62] has been criticized for not taking into account important life history or taxonomic variables [63], and plants may be particularly difficult subjects for calculating minimum viable population sizes [64]. Although effective population sizes can range from around one-tenth to one-third as a proportion of census size [65], a population consisting of around 200 adult trees in Singapore is likely demographically and stochastically vulnerable and hence may not be adequate for long-term viability despite the overall positive genetic data [11], [66].

The higher than expected genetic diversity we detected may be explained by demographic as well as ecological factors. In terms of demographics, a larger number of pre-fragmentation trees than expected may remain in the extant adult population. Hence, a high proportion of Singapore’s pre-deforestation genetic diversity may have been retained to the present day. For example, Singapore’s largest and presumably oldest primary forest trees are protected from lightning by conductors, leading to increased longevity. Conductors have been installed on many trees in our study area, and several mature *K. malaccensis* have had their conductor rods hit by lightning (E.L. Webb, pers. obs.). In addition, the presence of a small number of large, adult *K. malaccensis* outside of primary forest habitat suggests persistence of pre-deforestation individuals within the degraded secondary forest and urban matrix.

Ecologically, high outcrossing rates also likely contribute to genetic diversity detected. *Apis* has been identified as a pollinator of *Koompassia* (cited in [34]) and the distances between adults are generally within the flight limits and foraging distances of native *Apis* species in Singapore (*A. cerana, A. andreniformis*, and *A. dorsata*) [67]. Our results therefore provide strong evidence that pollination may aid tree species’ resilience in a degraded landscape [68], see also [23]. Hence, the ecology of the pollination vector is a critical determinant of whether pollen dispersal mechanisms can be maintained after habitat loss (and/or fragmentation). In the case of *K. malaccensis*, two key ecological aspects of pollination may be contributing to the high outcrossing rates of *K. malaccensis*. First is that *A. dorsata* forms colonies that may nest preferentially in *K. malaccensis*; secondly, *A. dorsata* is a highly mobile insect, capable of pollen transfer during foraging bouts or colony migrations of several km (R. Corlett pers. comm.). Other tree species with less mobile pollinators are unlikely to exhibit the same level of resilience to habitat loss as we have found with *K. malaccensis*.

## Conclusions

Our study stands in contrast to predictions of genetic erosion in formerly abundant species that have sustained rapid and dramatic population declines. However, long-lived species such as trees may need centuries for the accumulated effects of habitat loss and reduced population size to reach a critical threshold, and the century-old extinction debt in Singapore is likely ongoing. Stochastic factors (such as tree falls or continued urbanization) could eliminate rare alleles (e.g. six alleles are represented by a single individual, respectively), and demographic factors such as limited primary forest habitat, lack of recruitment in secondary forest, and a high seedling mortality rate may contribute to a potential extinction debt in the future.

Conversely, the continued functionality of insect pollinators implied by the data may help maintain viability of this small population by increasing the likelihood that the majority of alleles present in the adults are passed via sexual reproduction to the next generation (simultaneously maintaining genetic diversity while reducing genetic drift and inbreeding). Hence, the positive genetic results for *Koompassia malaccensis* in Singapore despite their demographic vulnerability suggest an “*Apis* rescue effect”: pollination provides a mechanism through which populations may retain resilience in the increasingly degraded and urbanized landscapes of Southeast Asia. However, the higher relatedness at short distances as well as the increasing SGS intensity in the recruit cohorts could indicate that Singaporean *K. malaccensis* may be undergoing a shift towards higher mating between (related) neighbours and a contracting neighbourhood size. Hence, the direction, frequency, and distance of pollen and seed dispersal within and between Singapore’s remaining primary forest fragments is crucial to determine the trends in population genetic dynamics resulting from habitat fragmentation.
